# Differential Fasting Plasma Glucose and Ketone Body Levels in GHRKO versus 3xTg-AD Mice: A Potential Contributor to Aging-Related Cognitive Status?

**DOI:** 10.1155/2017/9684061

**Published:** 2017-05-30

**Authors:** Chelsea M. Griffith, Lauren N. Macklin, Andrzej Bartke, Peter R. Patrylo

**Affiliations:** ^1^Department of Physiology, Southern Illinois University School of Medicine, Carbondale, IL 62901, USA; ^2^Center for Integrated Research in Cognitive and Neural Sciences, Southern Illinois University, Carbondale, IL 62901, USA; ^3^Division of Geriatrics Research, Department of Internal Medicine, Southern Illinois University School of Medicine, P.O. Box 19628, Springfield, IL 62794-9628, USA; ^4^Department of Anatomy, Southern Illinois University School of Medicine, Carbondale, IL 62901, USA

## Abstract

Cognitive function declines with age and appears to correlate with decreased cerebral metabolic rate (CMR). Caloric restriction, an antiaging manipulation that extends life-span and can preserve cognitive function, is associated with decreased glucose uptake, decreased lactate levels, and increased ketone body (KB) levels in the brain. Since the majority of brain nutrients come from the periphery, this study examined whether the capacity to regulate peripheral glucose levels and KB production differs in animals with successful cognitive aging (growth hormone receptor knockouts, GHRKOs) versus unsuccessful cognitive aging (the 3xTg-AD mouse model of Alzheimer's disease). Animals were fasted for 5 hours with their plasma glucose and KB levels subsequently measured. Intriguingly, in GHRKO mice, compared to those in controls, fasting plasma glucose levels were significantly decreased while their KB levels were significantly increased. Conversely, 3xTg-AD mice, compared to controls, exhibited significantly elevated plasma glucose levels and significantly reduced plasma KB levels. Taken together, these results suggest that the capacity to provide the brain with KBs versus glucose throughout an animal's life could somehow help preserve cognitive function with age, potentially through minimizing overall brain exposure to reactive oxygen species and advanced glycation end products and improving mitochondrial function.

## 1. Introduction

Aging is associated with a decline in cognitive function. During “normal” aging, individuals can either maintain cognitive performance or exhibit cognitive decline accompanied by a decrease in cerebral metabolic rate (CMR). Moreover, with pathological aging (i.e., with Alzheimer's disease (AD)), the degree of cognitive decline and decrease in CMR is even greater [1–3]. During both “normal” and pathological aging, this decrease in CMR appears to actually precede structural changes and the decline in cognitive function.

The antiaging manipulation known as caloric restriction (CR) has been shown to extend life-span in a diversity of species [4–6]. Further, it is generally believed that CR can preserve or improve cognitive function during aging with studies showing that (1) long-term reductions in energy intake can enhance cognitive performance in rats and mice [7, 8], (2) verbal memory is improved in elderly humans with a 30% reduction in caloric intake [9], (3) age-related deficits in learning and motor coordination are reduced by CR in rodents [10–12], and (4) AD-associated pathogenesis and cognitive decline are minimized or improved in the 3xTg-AD mouse model under caloric restriction [13]. However, the effect of CR is somewhat controversial since several other studies have reported no beneficial effects on spatial learning in both rats and mice [14–16], and one study using rats even reported a worsening of cognitive function despite increased longevity [17]. While the existing reason for this discrepancy in the literature is unknown, differences in the background strain, genotype, age of the animals used in the study, the duration of the treatment, or the specific diet used (e.g., differential composition of medium chain fatty acids [18]) could play a role. In this regard, it is interesting to note that recent experiments using multimetric neuroimaging have shown that CR decreases glucose uptake and lactate levels in the central nervous system (CNS) while KB levels are increased [19]. Thus, the antiaging benefits of CR may be due in part to this shift in metabolic phenotype, although further experiments are required that directly test this hypothesis. This idea however does gain some support from recent studies which demonstrate that ketone body supplementation can improve cognitive function in mild cognitive impairment (MCI) and AD [20]). Since the majority of nutrients utilized by the central nervous system (CNS) are derived from the periphery, we tested the hypothesis that the capacity to regulate peripheral glucose levels and produce KBs differs in animals that exhibit successful versus unsuccessful cognitive aging. Specifically, is peripheral glucose regulation enhanced in growth hormone receptor knockout (GHRKO) mice? GHRKO mice (GHR−/−) exhibit an extended life-span and do not show cognitive decline with age compared to controls, as assessed with the inhibitory avoidance task [21]. Further, is peripheral glucose regulation impaired in the 3xTg-AD mouse model of AD (3xTg-AD)? 3xTg-AD mice exhibit a decline in performance on numerous tasks with age [22], and their decrease in hippocampal-dependent cognitive function is associated with a decrease in CMR (i.e., glucose utilization) [23]. Conversely, could the degree of KB production be elevated in GHRKO mice yet decreased in 3xTg-AD mice? To test this hypothesis, animals were fasted for 5 hours with their plasma glucose and KB levels subsequently measured and comparisons made between each mouse model and their respective controls.

## 2. Materials and Methods

### 2.1. Animals

Male GHRKO (GHR−/−) mice (4–6 months) and wild-type or heterozygote littermate controls (GHR+/?) as well as 3xTg-AD mice (4–6 months) and wild-type controls were used for the experiments examining fasting plasma glucose and KB levels; all animals came from local breeding colonies at Southern Illinois University School of Medicine, Springfield and Carbondale, IL, respectively. Although male and female mice can exhibit differences in their metabolic profiles and thus different plasma glucose and ketone body levels [24, 25], male mice were exclusively used in this study to minimize any potential confound associated with metabolic changes seen in females during the different stages of the oestrus cycle [26]. The GHRKO colony was originally established by crossing 129Ola and BALB/c N (GHR+/−) mice with mice derived from crosses of C57BL/6 and C3H/J strains and has been maintained on this heterogeneous genetic background to more closely resemble a natural population. GHRKO mice were identified versus controls due to their reduced body size, weight, and length. 3xTg-AD mice were created by expressing mutated human amyloid precursor protein (hAPP) and human hyperphosphorylated tau primarily in the CNS through the use of a Thy1.2 promoter. These mice were subsequently mated to a PS1 mutant line to create the triple mutant [27] and thus are on a mixed C57BL/6J and C129 background. Wild-type controls were on the same background. All animals were housed in their respective vivariums and were kept on a 12 hr/12 hr light-dark cycle with Purina rodent chow (fat—13.5%, protein—28.5%, and carbohydrates—58%) and water provided ad libitum. All experiments were approved by the Institutional Animal Care and Use Committees (IACUC) at the respective sites and comply with the guidelines set forth by the National Institutes of Health.

### 2.2. Measuring and Comparing Plasma Glucose/Ketone Levels

To examine the periphery's capacity to regulate plasma glucose levels and produce KBs in GHRKO and 3xTg-AD mice, versus respective controls, mice were fasted for 5 hours with blood glucose and KB measurements subsequently made in duplicate using Precision Xtra glucometers (Abbott, Abbott Park, IL) and blood glucose or blood *β*-ketone strips (Abbott, Abbott Park, IL), respectively. Since background strain can dramatically affect peripheral metabolic function [28–30], statistical comparisons were only made between groups on the same background (i.e., GHR−/− versus GHR+/?; 3xTg-AD versus 129/C57BL6 wild types) using unpaired *t*-tests (Prism GraphPad 4.1, San Diego, CA).

## 3. Results

**Figure 1:**
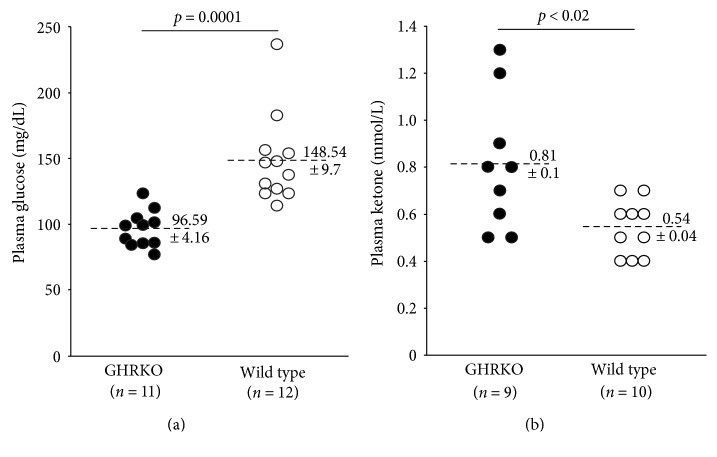
Fasting plasma glucose and ketone levels in GHRKO mice and controls. (a) GHRKO mice exhibited significantly lower plasma glucose levels compared to controls following a 5-hour fast. (b) GHRKO mice exhibited significantly greater plasma ketone levels compared to controls following a 5-hour fast.

**Figure 2:**
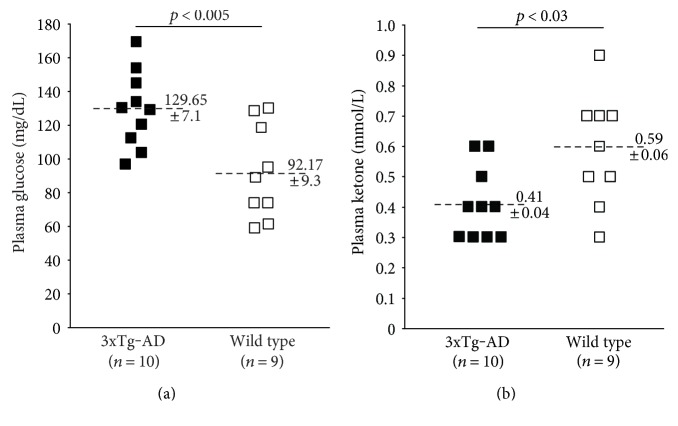
Fasting plasma glucose and ketone levels in 3xTg-AD mice and controls. (a) 3xTg-AD mice exhibited significantly greater plasma glucose levels compared to controls following a 5-hour fast. (b) 3xTg-AD mice exhibited significantly lower plasma ketone levels compared to controls following a 5-hour fast.

As shown in Figure 1, following a five-hour fast, GHRKO mice exhibit significantly decreased plasma glucose levels compared to controls (*p* = 0.001). Conversely, their plasma KB levels were significantly elevated (*p* < 0.02). In contrast, 3xTg-AD mice showed the exact opposite (Figure 2). Specifically, plasma glucose levels were significantly elevated in 3xTg-AD mice compared to controls (*p* < 0.005) while plasma KB levels were significantly decreased (*p* < 0.03).

## 4. Discussion

Among the most common neurological changes seen with age is a decline in cognitive function. Hippocampal-dependent learning and memory can be compromised with age in humans, nonhuman primates, and rodents [31–33]. Data suggest that this aging-related decline in hippocampal function is associated with altered bioenergetic properties. Specifically, modern imaging techniques reveal an aging-related decline in energy metabolism in the hippocampal formation of individuals undergoing “normal” as well as pathological aging [34–37], and the degree of hypometabolism observed (i.e., reduced glucose utilization) correlates with the extent of cognitive decline. While glucose is the preferred metabolic substrate of neurons and astrocytes in the adult CNS, a variety of alternative substrates including monocarboxylates (i.e., lactate, pyruvate, and acetate) and KBs (i.e., *β*-hydrobutyrate, acetoacetate, and acetone) can be oxidized to generate ATP. Further, monocarboxylates [38–41] and KBs [39, 41] can maintain synaptic efficacy and plasticity, the basis of cognitive function. In this regard, caloric restriction (CR) has been shown to cause decreased glucose uptake, decreased lactate levels, and elevated KB levels in the CNS [19]. Taken together, these data suggest that a reduction in glucose utilization and an increase in KB utilization could contribute to cognitive preservation with age. Consequently, since the CNS primarily derives it nutrients from the periphery, this study examined whether the regulation of plasma glucose and KB levels is altered early on in the life-span of GHRKO mice which exhibit cognitive preservation and the 3xTg-AD mouse model which develops cognitive impairment. The main findings of our current study were (1) as early as 4–6 months of age, GHRKO mice, compared to controls, exhibit significantly decreased fasting plasma glucose levels and elevated KB levels while (2) 3xTg-AD mice exhibit significantly elevated plasma glucose levels and significantly reduced KB levels compared to controls.

These data suggest that the capacity of the CNS to intermittently switch metabolic utilization from glucose to KBs throughout a host's life-span could positively impact age-related cognitive status. Specifically, in GHRKO mice, the reduction in plasma glucose levels coupled with elevated KB levels following fasting suggests that the CNS in GHRKO mice is intermittently more dependent on ketone bodies to maintain “normal” synaptic function. In contrast, in 3xTg-AD mice, the reduction in KB production and elevation of plasma glucose suggest an enhanced dependence of the 3xTg-AD CNS on glucose utilization. While cognitive function was not compared in the current study, due to cognitive preservation in GHRKO and 3xTg-AD mice at this early time point [21, 42], it is interesting to note the variability observed in plasma glucose and ketone body levels within an individual group. This raises the question of whether this variability in metabolic phenotype within a group could account for the variability observed in cognitive performance within either GHRKO, 3xTg-AD, or control groups with age. The reason for this difference in the capacity of the periphery to regulate plasma glucose and KB levels in GHRKO and 3xTg-AD mice is unknown, although one parsimonious explanation is that the capacity of the liver to undergo gluconeogenesis and beta oxidation differs in these mouse models. Further investigation is however required to directly test this postulation. Regardless, both a decrease in glucose dependence and an increase in KB utilization by the CNS could potentially contribute beneficial effects.

Cellular glucose utilization leads to the generation of reactive oxygen species (ROS) as well as advanced glycation end products (AGEs). During both “normal” aging and diabetes, the two leading risk factors for AD, AGEs are known to accumulate in the plasma and in various tissues at an accelerated rate [43, 44]. The main pathological consequence of AGEs interacting with their receptor (RAGE) is the induction of intracellular reactive oxygen species (ROS) [45]. Subsequently, ROS can lead to oxidative stress and sustained inflammatory responses, which could ultimately translate into irreversible cell damage, slow degeneration, and eventual cell death or synapse loss [46]. Thus, decreasing the metabolic dependency of glucose throughout a subject's life-span could in turn reduce the amount of oxidative damage and lead to successful cognitive aging.

Enhanced KB utilization throughout a subject's life-span could also contribute to cognitive preservation. First, *β*-hydroxybutyrate (BHB), the primary KB, can inhibit histone deacetylases [47] and also increase cytoplasmic acetyl CoA [48] which in turn subsequently increases histone acetyltransferase activity. Both of these changes can result in increased gene transcription contributing to longevity [45] and the formation of long-term implicit and explicit memory [49, 50]. Second, BHB can also decrease inflammation which results in a reduction in ROS and mitochondrial oxidative damage, thereby improving mitochondrial efficacy [51, 52]. Mitochondrial dysfunction has been linked to cognitive decline in numerous neuropathological conditions [53–55], and experimentally disrupting mitochondrial respiratory complex activity can impair synaptic efficacy and plasticity [56].

KBs can also directly impact AD-related pathogenesis and cognitive dysfunction. Treating animal models of AD with ketone esters results in elevated plasma KB levels and has been shown to slow or prevent AD pathology and improve cognitive performance [57]. Similarly, in a multicenter clinical trial with AC-1202 treatment, a drug that increases plasma BHB levels, improvement in Alzheimer's disease assessment scale (ADAS) cognitive scores was observed [58]. Further elevated plasma and brain ketone body levels could also affect AD via a variety of other mechanisms including (1) the blockade of A*β*_1–42_ (the toxic form of *β*-amyloid) entry into neurons which can decrease oxidative stress and amyloid burden [59] and (2) neuroprotection against *β*-amyloid toxicity possibly by decreasing ROS production and decreasing BACE1 expression, the rate-limiting enzyme in A*β*_1–42_ production [60]. These beneficial effects of BHB on AD pathogenesis and cognitive decline are intriguing given our data demonstrating a decreased capacity of the periphery to produce/provide KBs following a brief fast in the 3xTg-AD mouse model. In this regard, it is interesting to note that while CNS glucose uptake/utilization is decreased in AD, there is no change in KB uptake/utilization [61]. This raises the question as to whether a reduced KB supply to the CNS throughout a subject's life-span could potentially contribute to AD pathogenesis. Interestingly, Klosinski et al. [62] recently reported that aged female mice, a model of postmenopausal females which exhibit the greatest risk for sporadic AD, exhibit a precipitous drop in plasma ketone bodies and a concomitant increase in brain ketone body levels that appears to be the result of white matter degradation (i.e., catabolism). Thus, it is possible that a decreased capacity of the periphery to provide ketone bodies to the brain as an alternative fuel source could trigger white matter catabolism. The degradation of white matter, a characteristic of AD that correlates with cognitive status [63], would in turn be able to provide fatty acids for astrocytic beta oxidation and consequently elevated brain ketone body levels in an attempt to provide fuel at the expense of the network connectivity. Thus, further investigation into whether plasma KB levels may also be decreased in humans with AD or those with an enhanced susceptibility to AD (i.e., those exhibiting major risk factors such as diabetes) is warranted.

In conclusion, this study demonstrated an association between altered plasma glucose and KB levels during an animal's life-span and their capacity to eventually maintain cognitive function with age. These results are also consistent with those of studies on the beneficial effects of CR on aging-related cognitive performance, since CR results in decreased CNS glucose and lactate levels yet elevated KB levels. Taken together, these data reveal the importance of metabolite production and utilization during aging and illustrate the need for additional studies that examine why and how these differences in metabolite regulation/production occur in GHRKO versus 3xTg-AD mice.
